# Depression and obesity, data from a national administrative database study: Geographic evidence for an epidemiological overlap

**DOI:** 10.1371/journal.pone.0210507

**Published:** 2019-01-08

**Authors:** Jean-Christophe Chauvet-Gelinier, Adrien Roussot, Jonathan Cottenet, Marie-Claude Brindisi, Jean-Michel Petit, Bernard Bonin, Bruno Vergès, Catherine Quantin

**Affiliations:** 1 Psychiatry Unit, Department of Neurosciences, Dijon University Hospital, France; 2 INSERM Research Center 866, Dijon, France; 3 Biostatistics and Bioinformatics (DIM), Dijon University Hospital, University of Burgundy-Franche-Comté, Dijon, France; 4 Inserm, CIC 1432, Dijon, Dijon University Hospital, Clinical Investigation Center, Clinical Epidemiology/ Clinical Trials Unit, Dijon, France; 5 Department of Endocrinology and Metabolic Diseases, Dijon University Hospital, Dijon, France; 6 Psy-DREPI Laboratory, EA7458, University of Burgundy-Franche-Comté, Dijon, France; 7 Biostatistics, Biomathematics, Pharmacoepidemiology and Infectious Diseases (B2PHI), INSERM, UVSQ, Institut Pasteur, Université Paris-Saclay, Paris, France; Western University, CANADA

## Abstract

**Background:**

Depression and obesity are two major conditions with both psychological and somatic burdens. Some data suggest strong connections between depression and obesity and more particularly associated prevalence of both disorders. However, little is known about the geographical distribution of these two diseases. This study aimed to determine if there is spatial overlap between obesity and depression using data from the entire French territory.

**Methods:**

Data for 5,627 geographic codes for metropolitan France were collected from the two national hospital databases (PMSI-MCO and RIM-P) for the year 2016. We identified people who were depressed, obese or both registered in the two public medico-administrative databases, and we assessed their location. In addition, a multivariable analysis was performed in order to determine geographic interactions between obesity and depression after controlling for age, sex, environmental and socio-economic factors (social/material deprivation, urbanicity/rurality).

**Results:**

1,045,682 people aged 18 years and older were identified. The mapping analysis showed several cold and hot regional clusters of coinciding obesity and depression. The multivariable analysis demonstrated significant geographic interactions, with an increasing probability of finding a high prevalence of obesity in regions with major depression (OR 1.29 95% CI 1.13–1.49, p = 0.0002) and an increased probability of finding a high prevalence of depression in regions with a high ration of obesity (OR 1.32, 95% CI 1.15–1.52, p<0.0001).

**Conclusion:**

Our study confirms the significant bidirectional relationships between obesity and depression at a group level. French geographic patterns reveal a partial overlap between obesity and depression, suggesting these two diseases can be included in a common approach. Further studies should be done to increase the understanding of this complex comorbidity.

## Introduction

In Western countries, depression and obesity are two highly prevalent medical conditions with major psychological and somatic burdens [1,2]. In France, depression is a fairly frequent disease with a prevalence of 7% per year on average, affecting 4 million people. In addition, data from the European Study of the Epidemiology of Mental Disorders [3] demonstrated that a person living in France was more likely to develop a mood disorder than someone in Germany, Italy or Spain, illustrating the importance of this question throughout the French territory. Obesity is another major public health issue in France where the prevalence has reached 15%, affecting nearly 9 million people. Recent reports have underlined the constant and significant increase of obesity which increased by an alarming 76% between 1997 and 2012 [4]. Both depression and obesity are major health issues which require particular attention in order to reduce the global burden. In terms of mechanisms, a growing body of literature sustains that depression and obesity share some epidemiological, clinical and biological pathways [5–9] in a bidirectional manner, with obesity increasing the risk of depression and depression increasing the risk of obesity in prospective studies. Factors such as inadequate health habits, shared biological disturbances (i.e. low-grade inflammation, HPA axis dysfunction, neuroendocrine disorders, brain disorders, gut-brain microbiota axis troubles) and common psychological determinants (e.g. early trauma, deprived environment or inadequate coping strategies) [10–12] are often related to the development of both depression and obesity [13]. Moreover, the long-term physical, psychological and social consequences of obesity and depression, such as cardiometabolic disorders, comorbid psychiatric disorders, poor social outcomes and major stigma, give the medical community a number of valid reasons to focus on these two disabling conditions [2,11,12]. Recent studies have indicated that specific genotypes might contribute to atypical depression features with an associated risk of overweight and obesity, suggesting the relationship between depression and obesity may require special attention to prevent comorbid conditions [14,15]. The clinical and biological interactions suggest that the two diseases could be included in a common approach to early detection and synergistic therapeutic strategies, at least for substantial clinical subtypes. Nevertheless, most public health programs do not develop common procedures and lead separate campaigns against obesity and mood disorders.

Through previous research, prevention policies and treatment guidelines have been independently conducted or established, partly due to the traditional mind/body dichotomy, but it now seems more appropriate to explore the bidirectional relationship between obesity and depression using various approaches [5]. To this end, geographic mapping stands as a helpful method for generating rapid graphic summaries of patterns of disease prevalence, contributing to a better understanding of underlying mechanisms and helping to identify high and low-risk populations and areas. In order to determine whether the clinical and fundamental relationship recently demonstrated in individuals might have some epidemiological correspondence in a group-level analysis, a spatial and community approach to regional prevalence of depression and obesity may be of interest. For instance, Voutilainen et al. demonstrated spatial relationships between chronic diseases (e.g. coronary heart disease, diabetes) and psychiatric disorders in relation to environmental factors like urbanicity or living conditions [16]. The same type of analysis highlighted a high-prevalence cluster of diagnosed diabetes in the south of the United States which was correlated to educational and ethnic factors [17]. Other studies have described the epidemiological, psychological and social characteristics of MDD (Major Depressive Disorders) or obesity in different countries or regions [18–20], but no joint analysis of a potential obesity-depression spatial overlap is available.

In the present study we performed an exploratory geographic analysis of obesity and depression rates in French hospitals based on French national health data (i.e. the PMSI-MCO, Programme de Médicalisation des Systèmes d’Information en Médecine, Chirurgie et Obstétrique and the RIM-P, Recueil d’Informations Médicalisées en Psychiatrie), in order to determine a potential geographical overlap. In France, several studies have been conducted thanks to the availability and the exhaustiveness of medico-administrative databases such as the PMSI-MCO/RIM-P [21–25]. After controlling for age, sex and environmental factors (i.e. material/social deprivation or urban/rural living conditions), we used these databases to identify obese and depressed patients treated in hospitals, and we assessed their location in order to determine if there was geographical overlap between depression and obesity. Such an approach may provide useful epidemiological information in addition to the existing data suggesting substantial clinical and biological overlap between depression and obesity.

## Materials and methods

### Source of data and selection criteria

PMSI-MCO is the main source of data regarding hospitalization in France (MCO for medical, surgical, gynecological-obstetric), and the RIM-P (P for Psychiatry) is the main source for French psychiatric hospitalization data. These databases collect all of the public and private hospital stays for each patient (inpatients and outpatients) and allow patients to be followed over time thanks to a unique and anonymous linkage number. In the present study, we assessed depressed or obese people registered in the public medico-administrative data of the PMSI-MCO and RIM-P in the year 2016.

Patients were identified with specific ICD-10 codes for obesity or depression. The zip code of residence is recorded in the PMSI and the RIM-P for each patient, making it possible to map the hospital prevalence of obesity and depression.

French zip codes designate administrative areas ranging from hundreds (villages) to hundreds of thousands of inhabitants (major cities). In this study, zip codes were used in relation to the geographic codes found in PMSI or RIM-P data in order to localize our selection of patients.

Duplication of in or outpatient stays was carefully avoided and only single individuals with obesity and/or depression were recorded in the database, irrespective of each hospital stay during the year 2016.

This study analyzed inpatients and outpatients, males and females aged 18 years and more hospitalized in 2016 for depression or/and obesity. The patients were identified as follows:

1. Patients hospitalized for obesity
  - from the PMSI-MCO database:
    - Patients with main or associated diagnosis of obesity (ICD-10 code E66).
2. Patients hospitalized for depression
  - from the PMSI-MCO database:
    - Patients hospitalized for depressive disorder as main diagnosis (ICD-10 codes F32, F33);
    - Patients hospitalized for a suicide attempt (ICD-10 codes X6*, X7*, X8* as associated diagnosis) and a depressive disorder (ICD-10 codes F32, F33 as associated diagnosis) during the same stay.
  - from the RIM-P database:
    - Patients with a main or associated diagnosis of depressive disorder (ICD-10 codes F32-F33).

We used the same algorithms as other French studies which were conducted using national PMSI and RIM-P data [23,26].

### Spatial analysis

Prevalence of in-hospital obesity and depression were calculated from PMSI geographic codes, indicating each patient’s zip code of residence (5,627 PMSI geographic codes in France). It was standardized for age and sex according to the direct method using national census data from the French national census institute, the INSEE (Institut National de la Statistique et des Etudes Economiques) as a reference. We used INSEE data from the 2013 census for the PMSI geographic codes; 2013 data were the most recent for decennial age categories. Prevalence rates are expressed for 100,000 inhabitants.

In order to investigate the spatial overlap between obesity and depression, the mapping presents standardized prevalence rates for each condition with 2016 PMSI geographic codes. The prevalence rates were presented in two classes for each disease:

1. lower than the national average prevalence;
2. higher than the national average prevalence.

Four classes of geographic codes were thus defined, following a High-High/High-Low/Low-High/Low-Low discretization. For example, High-High means that prevalence rates of both obesity and depression are higher than the national average prevalence.

All spatial analyses were performed using GIS (Geographic Information System) MapInfo 11.0 and statistical analyses were performed with SAS 9.4.

#### Level of urbanization

We categorized the PMSI geographic codes according to the geographic areas established by the INSEE [27], which classifies urban areas according to the level of urbanization and the number of jobs held in the area. We aggregated the data for the PMSI geographic codes and retained 4 categories: major urban centers, the suburbs of major urban centers, small and mid-sized centers and rural areas.

#### Material and social deprivation index

A deprivation index was built using several social and economic measures such as unemployment rate, socio-economic level, diploma level, immigration rates, and income tax. Variables relative to socio-residential factors were generated from French census data [28] and household income data from 2013 [29]. We set our scores according to the average of the data sourced from the municipalities that make up each residence code in the PMSI.

The deprivation index was created from the combination of two measures of the socio-residential environment:

- Social deprivation, measured with standardized scores of unemployment, blue collar workers, people with no diploma or only a middle school diploma, and immigrants.
- Material deprivation, measured with the standardized score of non-taxable households.

The two scores were divided into three classes by taking -1 and +1 standard deviation as the borders of the distribution and then crossed so as to create a bidimensional scale following Pampalon’s "material and social deprivation index" model [30]. Five levels of population deprivation were identified according to the geographic code ranking for the two scores: 1) most advantaged 2) national average 3) material deprivation 4) social deprivation 5) most deprived.

Level 1 (class one for the two scores) designates the least deprived population for the two scores, and level 5 (class 3 for the two scores) designates the most deprived population. For level 3, the material disadvantage score was class 3, but there was no social disadvantage. On the contrary, in level 4 the social disadvantage was in class 3, but there was no material disadvantage. Level 2 of the deprivation index was organized according to the average of the two scores, where disadvantage was classified as 1 or 2, with at least one category reaching class 2.

### Multivariable analysis

A logistic regression analysis was performed to assess the relationship between the standardized prevalence rates of obesity and depression (by tertile) and the influence of environmental and socio-economic determinants. The independent factors entered into the multivariate model were the deprivation index, the level of urbanization, and depression (when obesity was the outcome) and obesity (when depression was the outcome).

## Results

**Fig 1:**
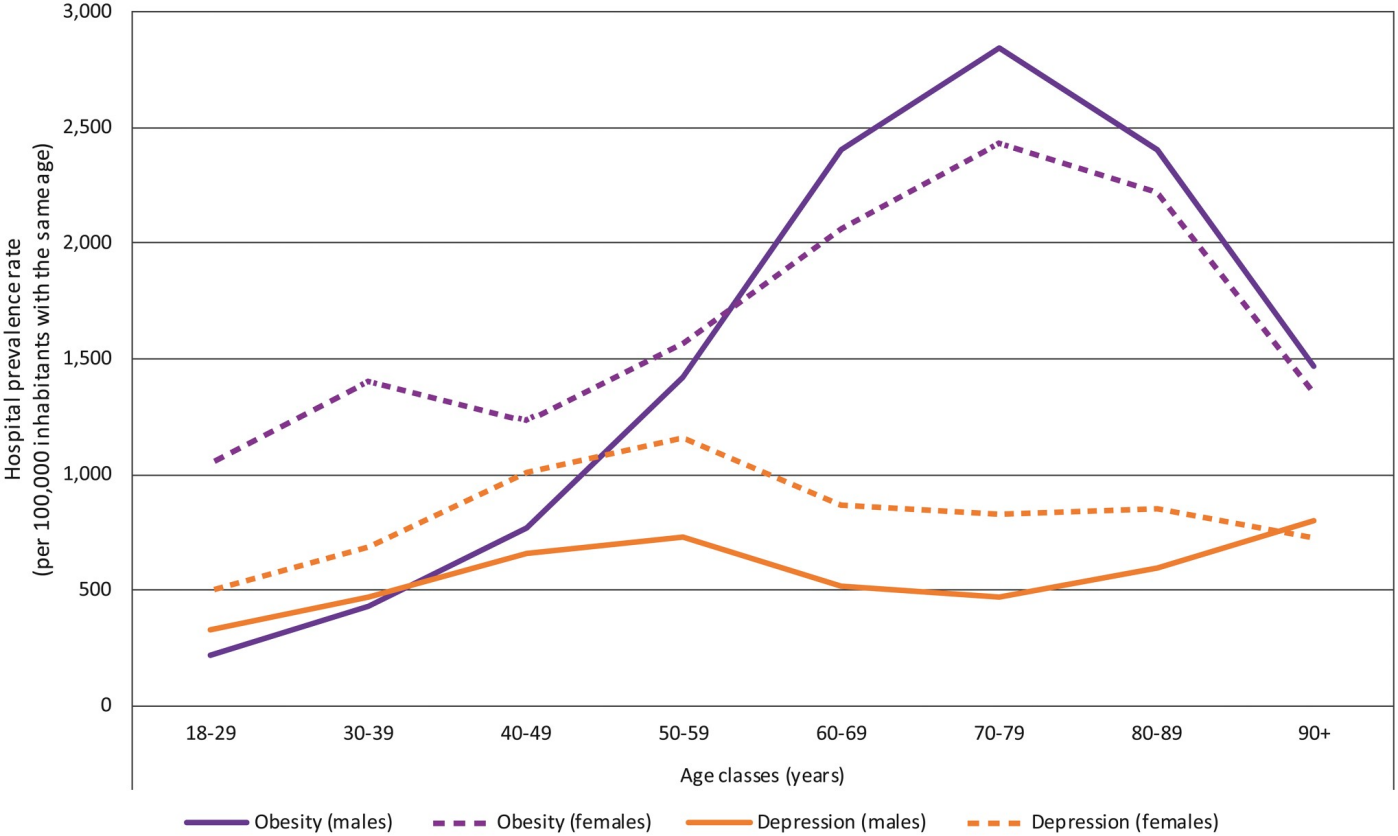
Age and gender distribution of hospital prevalence rates for obesity and depression.

The analyses were performed on 1,045,682 people identified as in- or outpatients with an ICD-10 code for obesity and/or depression in the national hospital database. We identified 707,680 hospitalized patients with an obesity-related diagnosis and 338,002 patients suffering from depression in a total of 5,627 French geographic areas. The general characteristics of the study population are summarized in Table 1 and in Fig 1. Obese patients were older than patients suffering from depression (mean age 58.2 ±17.4 vs. 52.5 ±17.2, p<0.0001).

**Table 1:** General characteristics of study population.

	Depression N = 338,002	Obesity N = 707,680	P value
**Age (years)**			
*Mean*	52.5 (±17.2)	58.2 (±17.4)	*<*.*0001*
**Sex (%)**			
*Women*	*63*.*48*	*58*.*72*	*<*.*0001*
*Men*	*36*.*52*	*41*.*28*	
**Deprivation Index (%)**			
*Most advantaged*	*8*.*22*	*6*.*92*	*<*.*0001*
*National average*	*67*.*05*	*64*.*29*	
*Material deprivation*	*4*.*30*	*4*.*81*	
*Social deprivation*	*12*.*33*	*14*.*65*	
*Most deprived*	*8*.*10*	*9*.*33*	
**Urban/Rural typology (%)**			
*Major urban centers*	*61*.*03*	*56*.*55*	*<*.*0001*
*Suburbs of major centers*	*19*.*53*	*23*.*36*	
*Small and mid-sized centers*	*9*.*59*	*9*.*11*	
*Rural areas*	*9*.*85*	*10*.*98*	

The peak in prevalence of obesity was 2,847 per 100,000 inhabitants aged from 70 to 79 years for males and 2,432 for females in the same class of age (Fig 1). The pick in prevalence of depression was 798 per 100,000 inhabitants aged 90 years and more for males and 1,165 for females between 50 and 59 years old (Fig 1).

More women than men were hospitalized for obesity-related (58.72% vs. 41.28%) and depression-related reasons (63.48% vs. 36.52%) (see Table 1). Patients hospitalized for depression were more likely to live in an advantaged or near-average socio-residential context than patients identified with a diagnosis of obesity. Indeed, compared to patients with depression, patients with obesity features were more likely to live in a context of material deprivation (4.81% vs. 4.30%, p<0.0001), social deprivation (14.65% vs. 12.33%, p<0.0001) and global precarity (9.33% vs. 8.10%, p<0.0001). Patients hospitalized for depression lived significantly more in major urban centers (61.03% vs. 56.55%) and less in the suburbs (19.53% vs. 23.36%). They were more likely to live in small and mid-size cities (9.59% vs. 9.11%) and less in rural areas (9.85% vs. 10.98%).

### Spatial analysis

**Fig 2:**
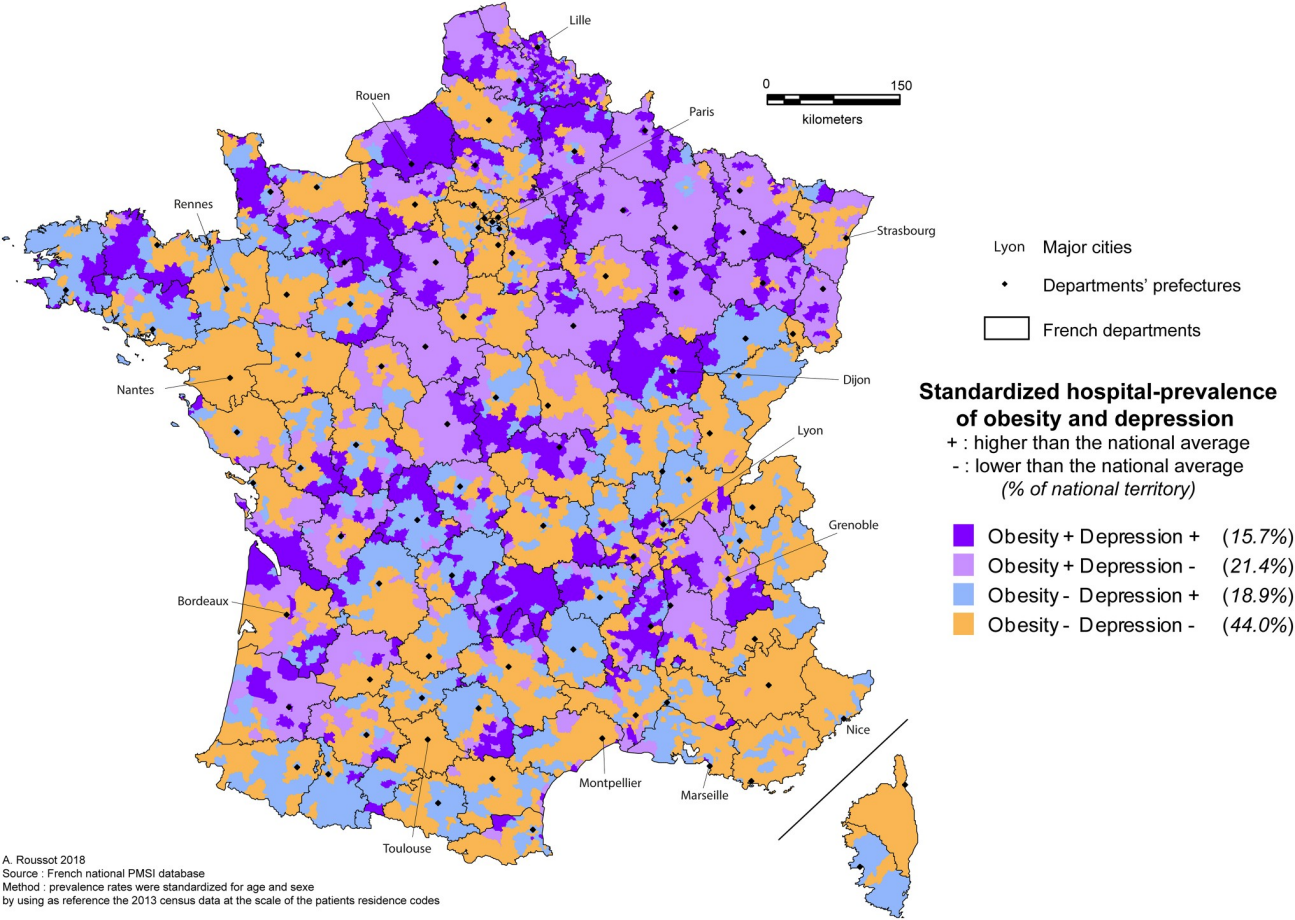
Hospital prevalence of obesity and depression.

The study highlighted some significant differences between France regions (Fig 2), with some clusters of co-occurrent over-representation (Brittany, Massif Central, North and North East part of France), and some clusters of under-representation (Pays de La Loire, Mediterranean belt, East of Auvergne-Rhône-Alpes area). Depression and obesity rates were simultaneously higher than the national average in 15.7% of the French territory, and depression and obesity were simultaneously lower than the national average in 44.0% of the territory, meaning that in 60% of the French territory the rates for the two conditions were conjointly above or below the national average.

### Multivariable analysis

We then analyzed the independent relationship between obesity and depression using the geographic codes.

The multivariable analysis revealed a highly significant mutual interaction between the geographic prevalence of obesity and depression (Table 2), with an increase in the risk of obesity in highly depressed regions (Odds Ratio (OR) of 1.295; 95% CI [1.128–1.486], p = 0.0002) (above average prevalence), but a protective effect in regions with moderate levels of depression (OR 0.704 [0.611–0.811], p<0.0001) (below average prevalence).

**Table 2:** Multivariable analysis of geographical interactions between obesity and depression.

Prevalence of obesity > 1,302 per 100,000 inhabitants		OR	95% CI		P value
**Prevalence of depression (tertiles)**	*Depression—(0–374)*	0.704	0.611	0.811	*<*.*0001*
	*Depression =*	Reference			
	*Depression + (> 559)*	1.295	1.128	1.486	*0*.*0002*
**Deprivation Index**	*Most advantaged*	0.444	0.341	0.579	*<*.*0001*
	*National average*	Reference			
	*Material deprivation*	1.484	1.217	1.809	*<*.*0001*
	*Social deprivation*	2.243	1.835	2.741	*<*.*0001*
	*Most deprived*	3.583	2.837	4.526	*<*.*0001*
**Urban/Rural typology**	*Major urban centers*	Reference			
	*Suburbs of major centers*	1.594	1.379	1.844	*<*.*0001*
	*Small and mid-sized centers*	1.373	1.109	1.699	*0*.*0036*
	*Rural areas*	1.492	1.267	1.757	*<*.*0001*

OR, odds ratio; CI, confidence interval

An analysis of the risk of depression as a function of the geographical prevalence of obesity showed similar results (Table 3). Geographically, the risk of depression was 1.323 [1.155–1.517] (p<0.0001) in regions with high obesity, and, conversely, the risk of depression was 0.724 [0.627–0.835] (p<0.0001) in regions with lower rates of obesity.

**Table 3:** Multivariable analysis of geographical interactions between depression and obesity.

Prevalence of depression > 559 per 100,000 inhabitants		OR	95% CI		P value
**Prevalence of obesity (tertiles)**	*Obesity—(0–851)*	0.724	0.627	0.835	*<*.*0001*
	*Obesity =*	Reference			
	*Obesity + (> 1*,*302)*	1.323	1.155	1.517	*<*.*0001*
**Deprivation Index**	*Most advantaged*	0.694	0.548	0.879	*0*.*0024*
	*National average*	Reference			
	*Material deprivation*	1.233	1.011	1.502	*0*.*0383*
	*Social deprivation*	1.157	0.941	1.422	*0*.*1669*
	*Most deprived*	1.648	1.308	2.078	*<*.*0001*
**Urban/Rural typology**	*Major urban centers*	Reference			
	*Suburbs of major centers*	0.765	0.662	0.885	*0*.*0003*
	*Small and mid-sized centers*	2.218	1.811	2.716	*<*.*0001*
	*Rural areas*	1.281	1.093	1.501	*0*.*0022*

OR, odds ratio; CI, confidence interval

In addition, the analysis demonstrated that areas of major material or social deprivation were associated with a high obesity rate: OR 1.484 [1.217–1.809] (p<0.0001) and 2.243 [1.835–2.741] (p<0.0001) respectively, and 3.583 [2.837–4.526] (p<0.0001) for people living in the most deprived socio-residential context. Concerning MDD, areas with material and global deprivation seemed to be more at risk of depression (OR 1.233 [1.011–1. 502], p = 0.0383 and OR 1.648 [1.308–2.078], p<0.0001), while individuals living in advantaged areas were less likely to develop depression (OR 0.724 [0.627–0.835], p = 0.0024). However, a high rate of depression was not associated with contexts of social deprivation.

Depression was also influenced by residence in urban or rural areas. Living in smaller rural cities (small and mid-size centers) was associated with depression (OR 2.218 [1.811–2.716], p<0.0001), and people living in the suburbs of major cities were less likely to be depressed (OR 0.765 [0.662–0. 885], p = 0.0003). Rurality was also associated to high rates of depression (OR 1.281 [1.093–1.501], p = 0.0022). All types of areas were associated with high rates of obesity with taking as reference the major urban centers.

## Discussion

This study is the first combined spatial approach to obesity-MDD comorbidity based on national hospital data from the PMSI-MCO and RIM-P databases from 5,627 French geographic codes, corresponding to the zip codes of patients’ residences. Three major findings have resulted from our work. First, several remarkable spatial comorbid obesity/MDD clusters were revealed, with regional hot spots where both obesity and depression were over-represented and cold spots where both obesity and depression were under-represented. Second, the multivariable analysis showed a significant bidirectional and positive spatial correlation between age-sex-standardized prevalence rates of depression and obesity, exhibiting a substantial geographical overlap between obesity and depression. Third, geographical and socio-environmental factors were associated with the obesity/depression comorbidity at a fine scale, showing the highly deleterious effect of major deprivation on both obesity and depression and illustrating the complexity of diseases with major bio-psycho-social determinants.

The present study suggests that the spatial prevalence of comorbid obesity/depression is not a random, widespread phenomenon in French regions, and confirms the existence of the frequent comorbid prevalence of obesity and depression in 60% of mainland France. While recent analyses provide a rationale for taking environmental factors into account in order to figure out the mechanisms behind psychiatric disorders [31], the statistical association between obesity and depression, and controlled for socio-economic factors, emphasizes socio-geographical bridges between depression and obesity, demonstrating a 33% mutual increase in MDD-obesity interaction. Our data are consistent with other epidemiological reports where pooled odds ratios of MDD in obese individuals ranged from 1.14 to 1.41 (using self-reported questionnaires or clinical diagnoses) [5,32,33]. So, we can consider that our study gives additional group-level information to mechanisms previously described in the common association between depressive disorders and obesity (i.e. poor health habits, sociodemographic factors, and various biological pathways). The first strength of this study is that the spatial design used a large national administrative database is a suitable approach concerning psychiatric disorders typically hard to screen in general population, especially because of major stigma [2]. The use of French geographic codes provides quite an accurate description of the spatial diffusion of obesity and depression treated in hospital facilities. The Mediterranean regions are spared from excessive levels of in-hospital obesity and depression, while the Massif Central, Brittany, Normandy, Northern and North-Eastern part of France suffer from an over expression of both obesity and depression. Individual habits such as diet may explain these differences in part—protective Mediterranean diet in some regions and saturated fat intake in others (Normandy, North/East of France). Furthermore, previous spatial analysis revealed that high prevalence of obesity in United States counties were linked to a low consumption of fruit and vegetables, and associated with less physical activity [18]. Still other reports have illustrated that imbalanced, fatty diets are associated with metabolic and inflammatory processes linked to both depression and obesity [10,34–36]. However, the absence of an absolute geographical overlap enhances the shared but very complex fundamental mechanisms involved in both depression and obesity, showing that individual evidence does not provide perfect correlation in a group approach. It emphasizes the necessity of specifying subclinical clusters within psychiatric disorders, such as determining distinctions between depression with and without obesity. Our obesity/MDD mapping of France does gives some useful information and represents an opportunity for health care programs to take a common approach to both conditions in order to stop the vicious circle of co-occurrence. Moreover, our detailed map reveals spatial risk clusters and gives, for the first time, a global mapping of the two conditions in France using hospital data. In addition, the multivariable analysis provides some interesting information about the social and environmental determinants associated with obesity and depression and confirms contextual impact in both conditions.

### Social deprivation and urbanicity

Mainly deprived areas, and areas with material deprivation to a lesser extent, were associated with depression, while the most advantaged areas were negatively associated with depression. However, social deprivation was not associated with depression. As mentioned above, we can speculate that people in the most advantaged regions adopt healthy lifestyles whereas people in most deprived areas have a poor diet and lack of physical activity, in addition to other psycho-social factors. A number of studies on depression have pointed out the complexity of socio-environmental factors, and it is worth noting that material deprivation has not been directly associated; several reports on well-being have underlined that the correlation between income and happiness is far from straightforward [37,38]. In our study, the most deprived geographic codes (i.e. both social and material deprivation) were strongly associated with increased depression, enhancing the need for progress in public policies in terms of socio-economic poverty reduction and development of health education programs. Regarding obesity, all deprived areas were linked to obesity regardless of material or social deprivation, which parallels existing literature [39,40]. Indeed, the relationship between obesity and poor socio-economic status or modest living conditions is well established. In the present study, we may assume that cold spots for obesity and depression (i.e. Pays de La Loire, East of Auvergne-Rhône-Alpes region, West and South-East France) are regions known for a quality of life, high socio-economic level and comparably low unemployment while hot spots for both comorbid obesity and depression are regions known for their socio-economic difficulties (North and East part of France), where global deprivation is significant. Previous reports have underlined the influence of socio-economic status on obesity [41,42], and the relationship is clearly present in France. The impact of regional socio-economic specificities likely limited the obesity/MDD mapping overlap. Indeed, if obesity seems highly related to low economic status as discussed previously, the prevalence of MDD depends on much more personal and environmental factors not directly related to socio-economic status, conveying different regional patterns for MDD and obesity. However, most deprived areas were associated with both obesity and depression, while individuals in most advantaged areas were less likely to develop both conditions. These crucial data require adjusted public policies for the most deprived zones in order to reduce geographical health disparities. On the one hand, very few French health campaigns have attempted to enhance public awareness of depression or to encourage screening and psychiatric care [43]. When developed, those rare programs focused mainly on mental health issues without addressing metabolic disturbances or other somatic outcomes possibly associated with depression [44]. In addition, there have been no specific programs for deprived regions or community groups. On the other hand, some public health media campaigns have focused on nutrition and healthy food, with a focus on the physical health consequences of a poor diet rather than a psychosocial approach. Yet recent reports [4] have stressed the need to develop health promotion in the most deprived French communities where obesity has increased dramatically over the last 10 years. Integrative biopsychosocial health programs appear appropriate for this context considering that motivational strategies are multifaceted and depend on mental determinants. Food intake and emotion share some fundamental pathways in terms of neuro-cognitive process, especially in people with high BMI. This has been demonstrated by several studies where healthy food behaviors were heightened by a focus on pleasure and tastiness rather than on a strict health rationale [45]. Thus, an integrative approach promoting health and positive emotions in communication and education may lead to healthier food habits in people with obesity, particularly in the most deprived areas. In this aim, our geographical and epidemiological analysis could inform the development of such integrative health programs in the most deprived areas, where both obesity and depression are highly prevalent.

Regarding urbanicity, though early reports underlined an association between population density and depression [46–48], the latest studies have dismissed this link, enhancing instead a specific risk in semi-rural regions and mid-sized urban centers in rural territories as illustrated here. These results appear to be in line with Reeves et al. and Breslau et al. who showed the deleterious aspects of rurality and small urban centers vs. large metropolitan centers [49,50], which might be due to the lack of psychiatric resources in small towns and rural areas. An older study conducted in England highlighted the link between suicides and the remoteness from large urban centers, concluding that individual isolation in certain rural areas might lead to major depression and suicide [51]. Concerning obesity, our study highlighted that the suburbs of major cities had an increased risk of high prevalence rates. Similarly, American studies have pointed out the negative effect of urban sprawl on obesity rates. People living in large suburbs may develop worse life habits, such as a sedentary lifestyle or more motorized transports [41,52,53]. This has been referred to as an “obesogenic environment” [54] even if the interactions between urban environment and obesity remain complex [55,56]. Recent data revealed that inhabitants of rural areas, where specific socio-residential risks like precarity may be associated to a lower access to health facilities, would be more at risk of developing overweight [57], which is in line with our study. In the United States, some data showed a similar geographical association between obesity and diabetes in geographic mapping, in spite of cultural specificities and differences [17,58]. So, the additional socio-environmental data concur with existing literature, and emphasize our main result, the partial but robust geographical overlap between obesity and depression.

### Age and gender differences

The demographic characteristics of our study population are similar to those generally reported in epidemiological surveys for both mood disorders and obesity. The depression peak observed in the present report corresponds to the over-representation of depression between 40 to 60 years, mainly in women, often described in mental health epidemiology [10]. As far as obesity is concerned, many reports have shown that the prevalence of obesity increases with age. In France, recent studies have demonstrated that obesity was twice as prevalent in the 55–74 age group than in the 18–39 age group [4]. Thus, our data are in line with existing epidemiological data.

The present study highlights some gender differences in terms of health care access. If depression rate in males and females is consistent with the standard epidemiology of MDD (females 66% vs. males 33%) [59–61], it is not the case for hospital prevalence of obesity. Indeed, our study demonstrates that women suffering from obesity are hospitalized much more frequently than men until the age of 60. This observation is similar to a previous study on health perceptions and underassessment of body weight in males [62]. Thus, while epidemiological reports describe a balanced sex ratio in obesity prevalence and more overweight men than women, it is worth mentioning that men and women have different beliefs and behaviors relating to weight issues. Overweight males are less likely to see themselves as overweight than females in the same situation. Furthermore, other reports demonstrated than men are underrepresented in weight-loss programs [63]. In addition to a health perception bias, men see most weight loss programs as unattractive or designed for women’s needs [64]. Similarly, there has been a marked gender difference in patients undergoing bariatric surgery (males 20% vs. females 80%) for several decades [65]. All these data illustrate the major psycho-behavioral obstacles that discourage men from taking charge of their weight problems. The very low rate of hospitalized males with obesity in the present study confirms a detrimental gender bias. An understanding of this bias should encourage the development of specific programs for men, from primary care to specialized nutritional services.

### Limitations

This study has several limitations. First, as we used aggregated data at the scale of French zip codes to evaluate the overlap between two pathologies and the potential association with several socio-residential indicators, our results may be subject to an ecological bias [66]. However, the object of this study was not to investigate individual propensity to develop both conditions but to explore spatial tendencies for a whole country. Several spatial studies using the same type of data and scale of analysis have demonstrated the robustness of this type of analysis [21,67,68]. Second, we could not explore obesity and depression in primary care, but our study probably identified the most severe cases of depression and obesity that required in-hospital health services. In addition, the obesity and depression rates demonstrated in this research are logically minor in comparison with French annual prevalence of both diseases [4,69] because of the in-hospital approach. However, we may think that in-hospital spatial distribution of prevalence rates might reflect the global spatial prevalence of the two diseases, irrespective of severity. Third, in this study we included overweight patients (25<BMI<30) but we are not sure that we could identify them exhaustively. This limitation might have led to an under-evaluation of metabolic dysregulations due to the ICD-10 code threshold. We may speculate that more comorbid depressed-overweight people would have been revealed by including all non-obese overweight patients, which would have probably strengthened the main statistical trends of the present study, i.e. the robust statistical association between obesity and depression. Fourth, our analysis was standardized for age and sex but did not take into account lifestyle or antidepressant medication, though those confounding factors were not strongly associated with obesity in depressed people in several reports. Indeed, epidemiological evidence supports the association of depression and obesity in both Western and non-Western countries, with a robust bidirectional relationship, independently of antidepressant medications [5,70]. In fact, weight gain related to psychotropic medications appears more to be linked to polygenic risk more than comorbid depression/obesity mechanisms [71]. Despite these limitations, other reports have demonstrated similar spatial correlations in linked pathologies (e.g. obesity, diabetes and stroke) [16,18], and we believe our study provides useful additional epidemiological data including group-level associations between obesity and depression which underline shared clinical and biological mechanisms.

## Conclusion

In conclusion, this novel geographical approach provides additional results regarding prevalent comorbidity between obesity and depression, confirming that a wider, community-level approach may be of interest in understanding pathophysiological mechanisms and community-level discrepancies. Our approach confirmed in fine detail the mutual interactions between obesity and depression, and underlined the average risk of worsening a primary condition with comorbid depression or obesity of 33 and 34%, respectively. No absolute spatial overlap was revealed but some highly co-morbid regions were identified, enhancing the major complexity of obesity/MDD relationships. This study invites stakeholders to envision a synergistic clinical and biological approach to these two medical conditions, and to be on the lookout for regional discrepancies in obesity/depression rates. Prevention and educational programs should be promoted in order to reduce obesity and depression in men and women, especially in certain high-risk areas highlighted in this report. A holistic, integrative approach is an important step to encourage prevention, which is preferable to medical treatment. Further studies are needed to explore the elements contributing to both obesity and depression and, in particular, regional differences.

## Ethics

This study was approved by the French national committee for data protection (Commission Nationale de l’Informatique et des Libertés, registration number 1576793) and was conducted in accordance with the Declaration of Helsinki. Individual written consent was not needed for this study.

## Supporting information

S1 Fig Sudy area (major French regions with the location of the zip codes). (TIF)

S2 Fig French spatial distribution of social/material deprivation and urban/rural typology. (TIF)
