# Supplementary figures and images for: Depression and obesity, data from a national administrative database study: Geographic evidence for an epidemiological overlap
Source: PLoS One. 2019 Jan 8;14(1):e0210507. doi: 10.1371/journal.pone.0210507 (PMC6324832; doi:10.1371/journal.pone.0210507)

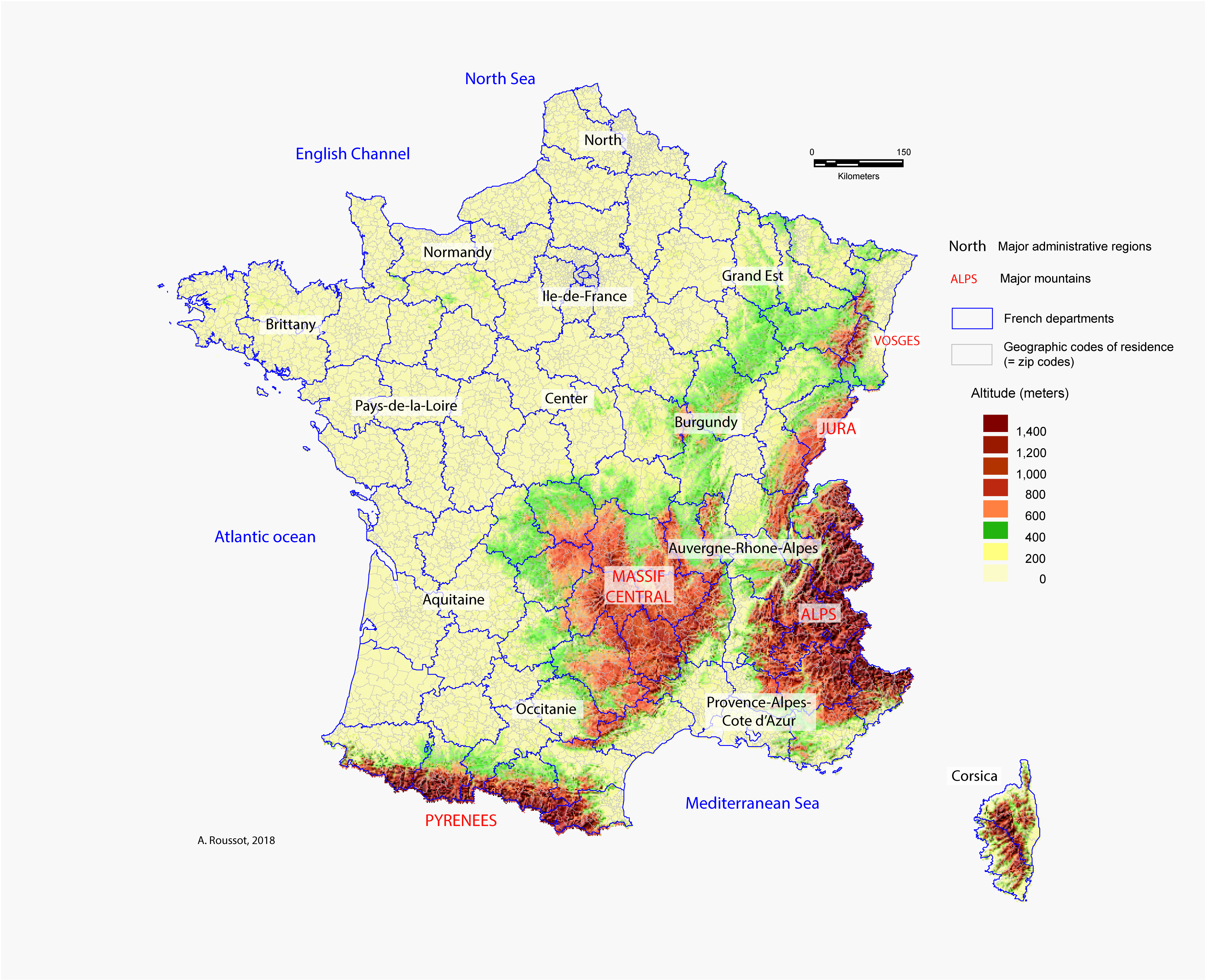

Supplement: S1 Fig — (TIF) [file pone.0210507.s001.tif]

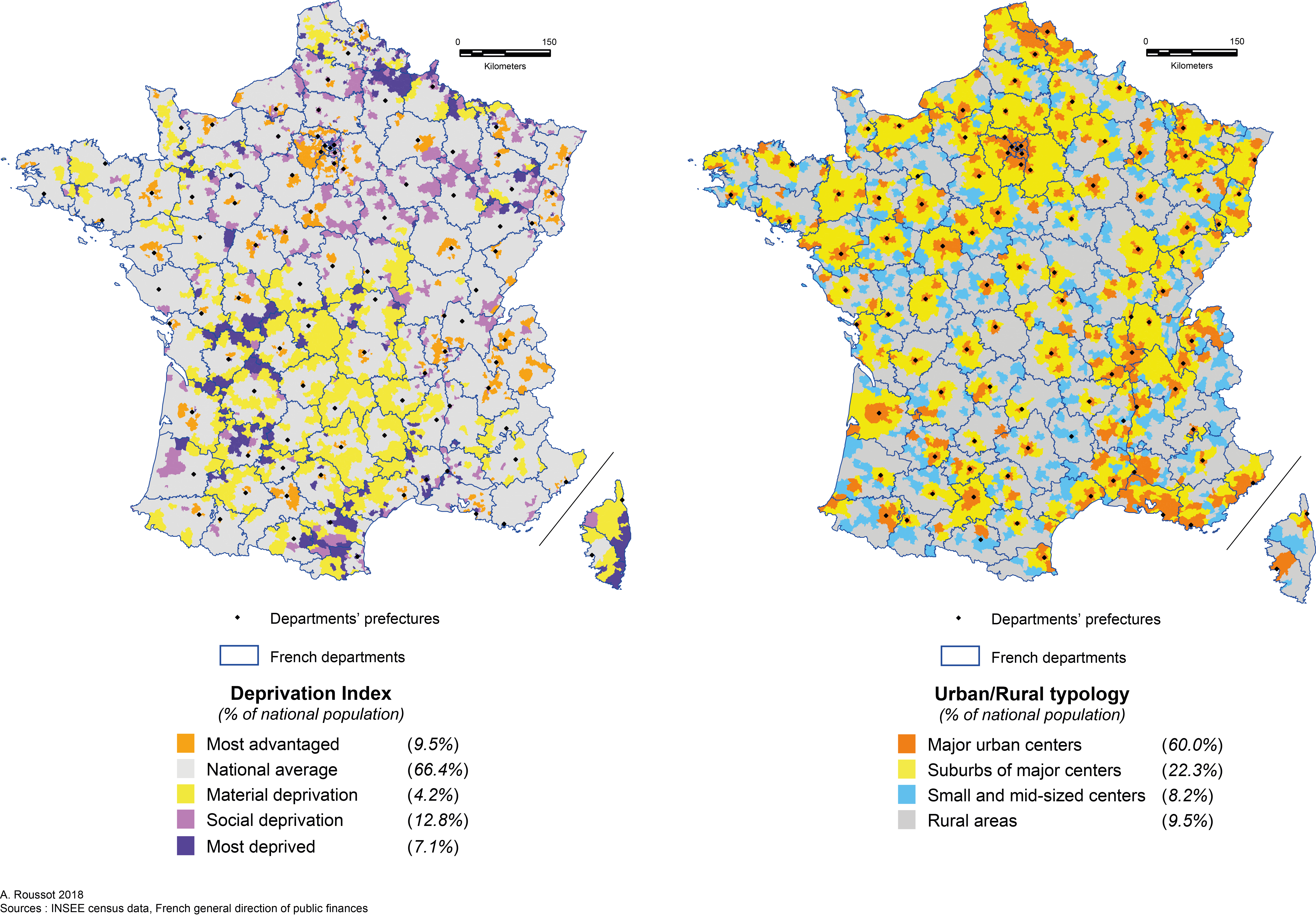

Supplement: S2 Fig — (TIF) [file pone.0210507.s002.tif]
